# Supplementary material for: Anti-Tumor Efficacy of PD-L1 Targeted Alpha-Particle Therapy in a Human Melanoma Xenograft Model
Source: Cancers (Basel). 2021 Mar 12;13(6):1256. doi: 10.3390/cancers13061256 (PMC8000940; doi:10.3390/cancers13061256)
Supplement: Supplementary file 1 [file cancers-13-01256-s001.pdf]

Table S1: Analysis of tumor proliferation**.** Slides from four different M113$^{PD\text{-}L1+}$ and M113^{WT} tumors were stained using anti-Ki67 or isotype control mAbs and counterstained with Hematoxylin. Cell quantification was performed using QuPath 0.2.3 software.

Tumor		Staining	Cell count			Positive cells	
**M113**	**n°**		**Total**	**Negative**	**Positive**	**%**	**Mean ± SD**
PD-L1+	1	Isotype control	46 104	45 685	419	0.91	0.95 % ± 0.16
	2		23 599	23 420	179	0.76	
	3		41 048	40 575	473	1.15	
	4		20 072	19 880	192	0.96	
	1	Ki67	43 929	11 761	32 168	73.23	70.33 % ± 3.61
	2		20 832	5 736	15 096	72.47	
	3		33 506	11 654	21 852	65.22	
	4		18 090	5 359	12 731	70.38	
WT	1	Isotype control	58 640	58 234	406	0.69	0.69 % ± 0.19
	2		68 795	68 437	358	0.52	
	3		33 642	33 323	319	0.95	
	4		69 960	69 549	411	0.59	
	1	Ki67	50 253	16 139	34 114	67.88	63.73 % ± 8.66
	2		61 410	21 611	39 799	64.81	
	3		29 618	8 606	21 012	70.94	
	4		67 083	32 667	34 416	51.3	

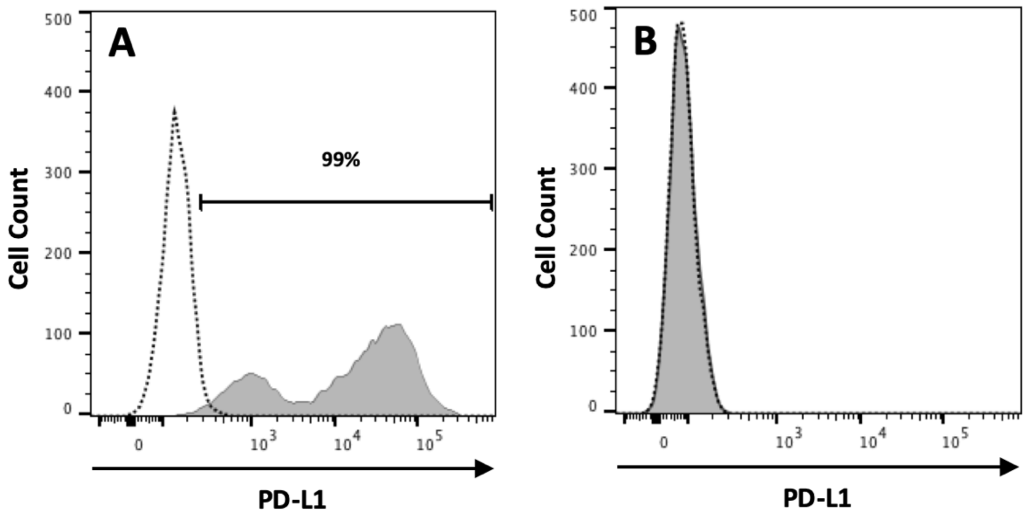

Figure S1: PD-L1 expression on M113$^{PD\text{-}L1+}$ and M113^{WT} melanoma cells *in vitro. In vitro* expression of PD-L1 was assessed by flow cytometry on M113$^{PD\text{-}L1+}$ (A) and M113^{WT} (B) cell lines, using a PE-conjugated anti-hPD-L1 mAb (gray histogram) or PE-conjugated mouse IgG2b isotype control (--- histogram). Flow cytometry was performed on a BD FACS CantoII™ system. Data are representative of more than 5 separate experiments. Expression appeared heterogenous on the cells, with 75% expressing high levels and 25% expressing low levels of PD-L1.

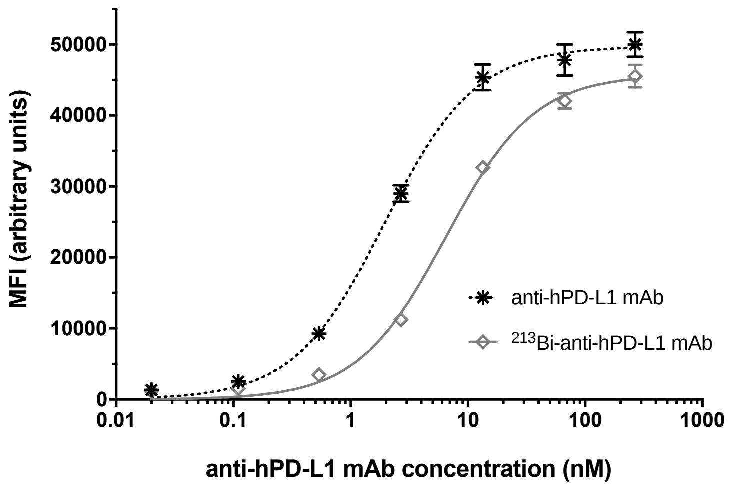

Figure S2: Binding affinity of unlabeled anti-hPD-L1 and ^{213}Bi-anti-hPD-L1 mAbs on M113$^{PD-L1++}$ melanoma cells. M113$^{PD-L1++}$ melanoma cells were incubated *in vitro* with increasing concentrations of unlabeled or radiolabeled anti-hPD-L1 mAbs followed by incubation with PE-conjugated polyclonal anti-mouse IgG Ab. After staining, cells were analyzed by flow cytometry using a FACSCanto II and FlowJo software. Kd was determined based on fluorescence intensity using prism graphpad software. Experiment was performed in duplicates and means $\pm$ SD are plotted.

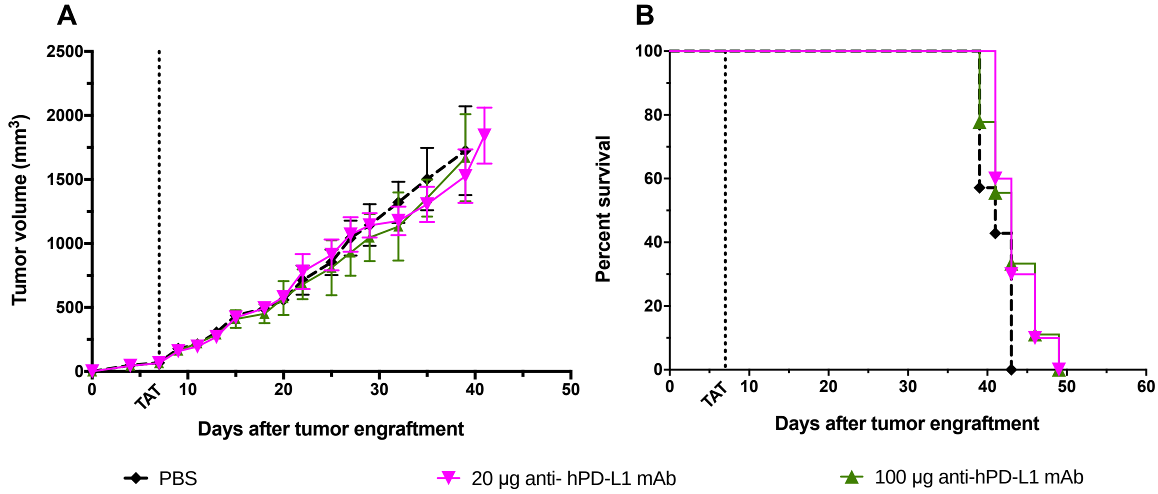

Figure S3: M113^{PD-L1+} melanoma xenograft tumor growth and survival after immunotherapy with unlabeled anti-hPD-L1 mAb**.** At day 0, NSG mice were grafted subcutaneously with 1×10^6 M113^{PD-L1+} melanoma cells. At day 7, treatment was performed by *i.v.* injection of 20 µg anti-hPD-L1 mAb (▼, n=10), 100 µg anti-hPD-L1 mAb (▲, n=9) or PBS for control animals (◆, n=7). (A) Tumor volume, represented by mean and SD, was determined sequentially from engraftment until until signs of tumor necrosis or volume reached 2000 mm^3 and animals were sacrificed. No difference was observed in tumor growth between the different groups. (B) Kaplan-Meier survival analysis. Treatment with 20 or 100 µg unlabeled anti-hPD-L1 mAb had no impact on survival (MS=43 days for both groups) compared to PBS control group (MS=41 days).
